# Successful treatment of aortic valve endocarditis caused by *Enterococcus casseliflavus*: a case report

**DOI:** 10.1186/s12879-021-06160-1

**Published:** 2021-05-18

**Authors:** Nobumasa Okumura, Takashi Watanabe, Satoshi Teranishi, Daisuke Suzuki, Takahiko Hashimoto, Kosuke Takahashi, Toru Hara

**Affiliations:** 1 Department of Respiratory Medicine, Anjo Kosei Hospital, Anjo-cho Higashi-Hirokute 28, Anjo City, Aichi Prefecture Japan; 2 Department of Cardiology, Anjo Kosei Hospital, Anjo-cho Higashi-Hirokute 28, Anjo City, Aichi Prefecture Japan; 3 Emergency Department, Anjo Kosei Hospital, Anjo-cho Higashi-Hirokute 28, Anjo City, Aichi Prefecture Japan; 4 Department of Infectious Diseases, Anjo Kosei Hospital, Anjo-cho Higashi-Hirokute 28, Anjo City, Aichi Prefecture Japan

**Keywords:** *Enterococcus casseliflavus*, Enterococci, Infective endocarditis, Intrinsic resistance

## Abstract

**Background:**

*Enterococcus casseliflavus* is rarely isolated from human specimens. To the best of our knowledge, there are no reports on its detailed treatment course and prognosis. Here, we present the first known case of *E. casseliflavus* endocarditis with a detailed treatment course.

**Case presentation:**

An 86-year-old Japanese woman was transferred to the emergency department with dyspnoea, wheezing, and lumbago. Her medical history included hypertension, chronic kidney disease, idiopathic interstitial pneumonia, and rectal carcinoma. Physical examination revealed expiratory wheezes and a diastolic murmur (Levine 2/6) at the 4th right sternal border. Chest radiography revealed bilateral interstitial opacities and slight cardiac dilatation. Transthoracic echocardiography demonstrated the presence of mobile vegetation with perforation, prolapse, and regurgitation of the aortic valve. With a suspicion of infective endocarditis, we started administering intravenous ampicillin/sulbactam. Thereafter, blood cultures identified *E. casseliflavus* through matrix-assisted laser desorption/ionisation time-of-flight mass spectrometry. The antimicrobial treatment was then switched to ampicillin plus gentamicin. The patient underwent aortic valve replacement on the thirteenth hospital day. She was administered intravenous ampicillin and gentamicin for 6 weeks. The patient was discharged 8 weeks after admission.

**Conclusions:**

Our case demonstrated that *E. casseliflavus* could cause infective endocarditis, which can be successfully treated with a 6-week regimen of ampicillin and gentamicin in combination with proper surgical treatment.

## Background

Enterococci are Gram-positive, facultatively anaerobic bacteria that are normal commensals of the human gastrointestinal tract [1]. However, they are known to cause hospital-associated infections such as bloodstream and urinary tract infections [1]. They also cause infective endocarditis and are estimated to account for approximately 10% of cases of infective endocarditis [2]. *E. faecalis* and *E. faecium* are the major species isolated from clinical sources in patients with endocarditis. Among patients with enterococcal endocarditis, *E. faecalis* is the causative organism in 97% cases, *E. faecium* in 1 to 2%, and other species in the remaining 1% [2]. *E. casseliflavus*, formerly known as *Streptococcus faecium* var. *casseliflavus*, was isolated for the first time in 1968 from a number of samples obtained from several plants [3]. *E. casseliflavus* was determined to be the same species as *E. flavescens* and was subsequently referred to as *E. casseliflavus/flavescens* [4]. It is currently part of the *E. gallinarum* group along with *E. gallinarum* [5]. *E. casseliflavus* is rarely isolated from human clinical specimens. It is known to cause blood stream infection, meningitis, and endophthalmitis in humans [5]. However, to the best of our knowledge, there are few reports on infective endocarditis caused by *E. casseliflavus*, and there are no detailed reports on the treatment course used, such as antibiotic regimen, dose, duration, and surgical intervention. We report the first case of *E. casseliflavus* endocarditis with a detailed treatment course.

## Case presentation

**Fig. 1:**
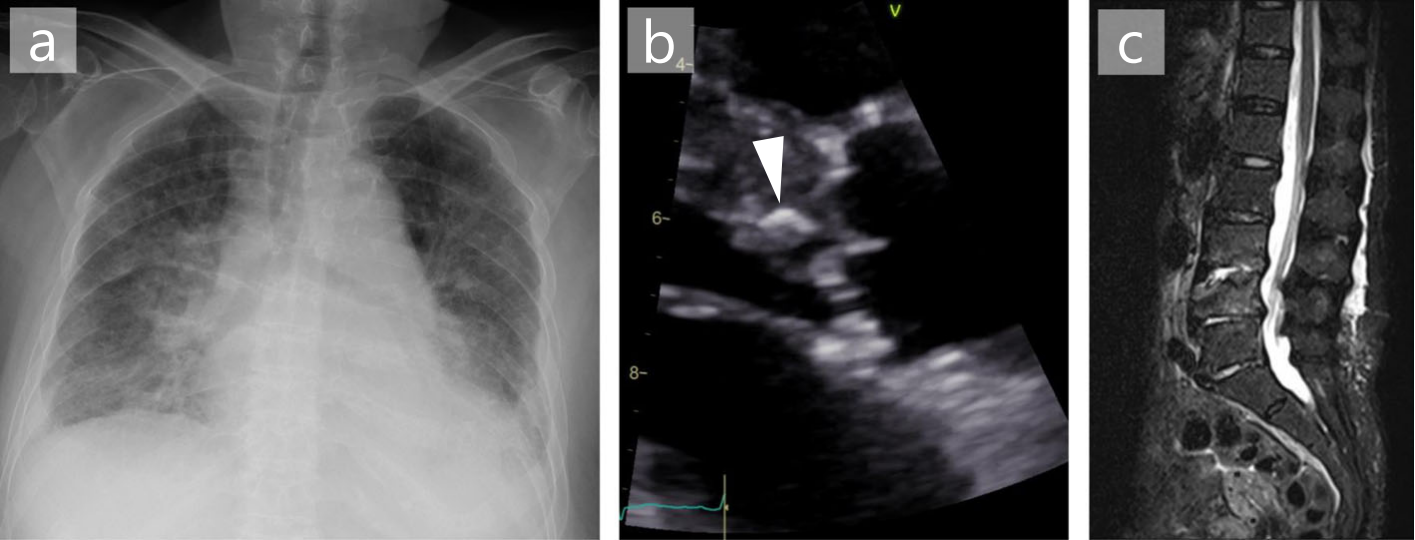
Diagnostic imaging of *Enterococcus casseliflavus* endocarditis. **a.** Chest radiograph showing bilateral interstitial opacities, pleural effusion, and slight cardiac dilatation; **b.** Transthoracic echocardiogram (parasternal long-axis view) showing aortic valve with vegetation (arrowhead); **c.** A lumbar MRI STIR image showing osteolytic lesion at the L3/4 level

An 86-year-old Japanese woman was transferred to the emergency department with rapidly progressive dyspnoea and wheezing. She also reported a five-month history of lumbago. Her medical history included hypertension, chronic kidney disease, idiopathic interstitial pneumonia, and rectal carcinoma (resected). She had never undergone heart valve surgery or dental treatment. On examination, she was afebrile, and her blood pressure was 165/70 mmHg, pulse 88 /min, and respiratory rate 16 /min with O_2_ saturation of 78% on room air. Physical examination was notable for expiratory wheezes and a diastolic murmur (Levine 2/6) at the 4th right sternal border. There was no evidence of rash or lumbar spine tenderness. Laboratory tests showed normocytic anaemia (haemoglobin level, 9.2 g/dL; mean cell volume, 91.5 fL), mild renal impairment (serum creatinine level 0.72 mg/dL, Cockcroft-Gault CCr 39.1 mL/min), inflammation (C-reactive protein level 4.61 mg/dL), and elevated brain natriuretic peptide level (319.7 pg/mL). Chest radiography revealed bilateral interstitial opacities, pleural effusion, and a slight cardiac dilatation (cardiothoracic ratio 64%, Fig. 1a). Transthoracic echocardiography demonstrated the presence of mobile aortic vegetation (9.5 × 4.3 mm in size, Fig. 1b) with perforation, prolapse, and regurgitation of the aortic valve. Magnetic resonance imaging (MRI) of the lumbar region revealed destruction of the disc structure and adjacent vertebrae at the L3/4 level (Fig. 1c). A brain MRI showed no evidence of cerebral infarction or aneurysm.

**Fig. 2:**
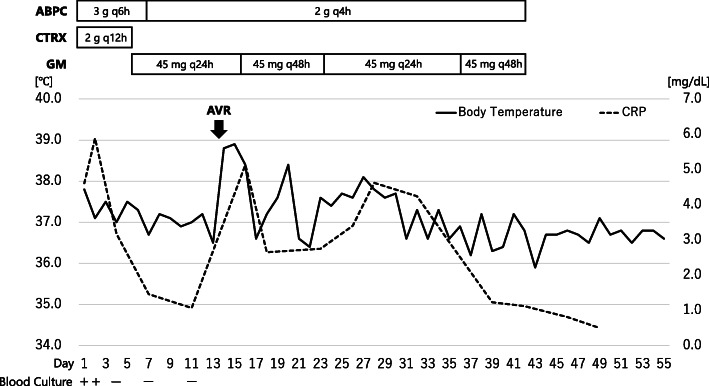
Timeline of clinical course**.** AVR: aortic valve replacement; CRP: C-reactive protein; ABPC: ampicillin; CTRX: ceftriaxone; GM: gentamicin; q6h: every 6 h

**Fig. 3:**
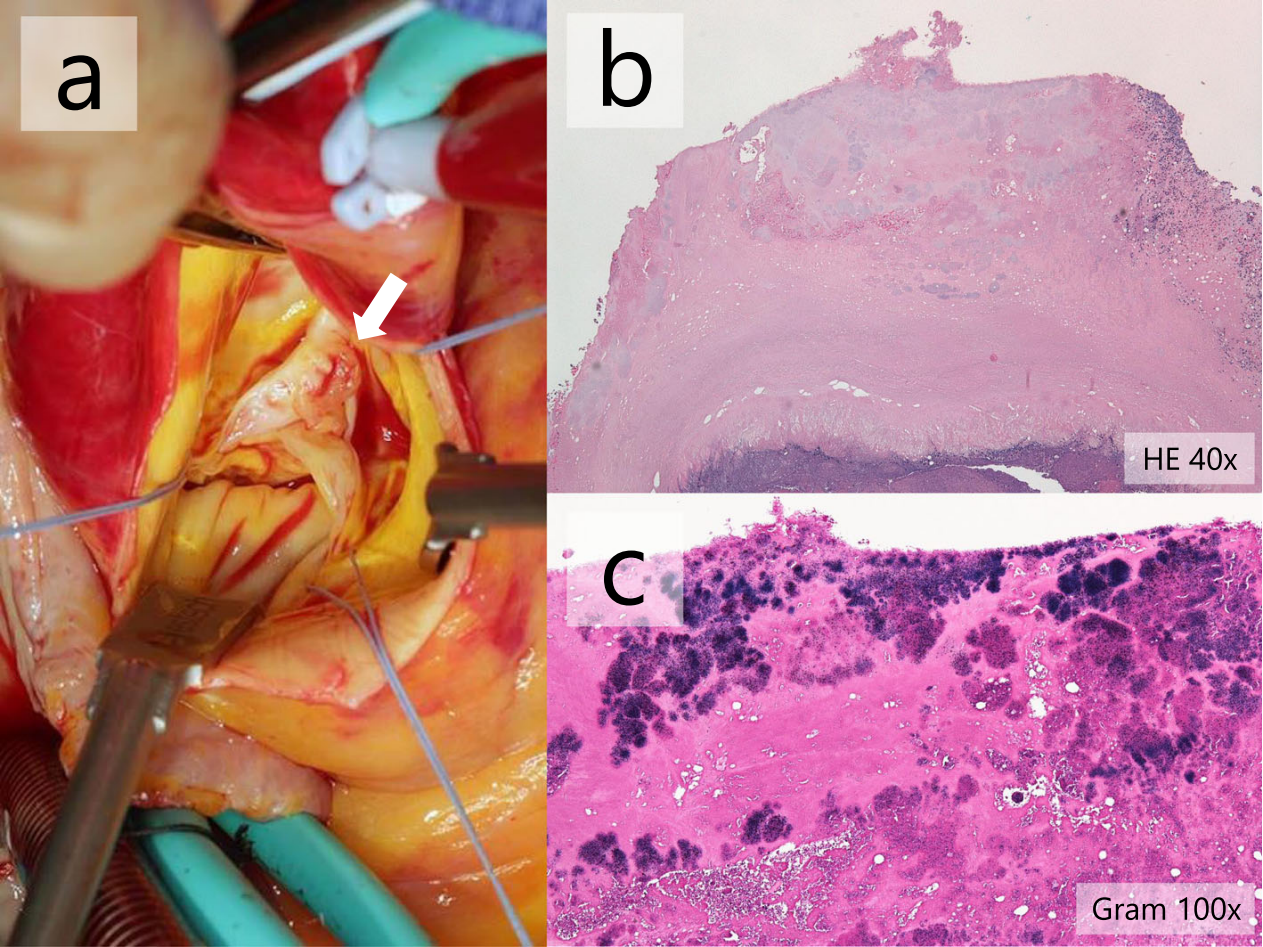
A picture and histopathological examination of the aortic valve. **a.** Intraoperative picture of the aortic valve with vegetation (arrow); **b.** Haematoxylin Eosin staining of the resected aortic valve showing nodular infiltration of lymphocytes and neutrophils into whole layers with fibrin precipitation and necrosis (× 40); **c.** Gram staining showing clusters of Gram-positive cocci on the surface (× 100)

Intravenous ampicillin/sulbactam, furosemide, and carperitide were administered based on a likely diagnosis of infective endocarditis, followed by pulmonary oedema and vertebral osteomyelitis. Gram-positive coccus, which was identified as *E. casseliflavus* through matrix-assisted laser desorption/ionisation time-of-flight mass spectrometry (MALDI-TOF MS; MALDI Biotyper, BRUKER, Billerica, MA, USA), was isolated from two sets of blood culture bottles on the day after admission. Soon after the causative microorganism became clear, we switched the antimicrobial regimen to ampicillin (3 g every 6 h) and ceftriaxone (2 g every 12 h, Fig. 2). Based on the broth microdilution method, the strain was susceptible to penicillin, ampicillin, and daptomycin (minimal inhibitory concentration 1, 1, 1 μg/mL, respectively) according to the Clinical and Laboratory Standards Institute guidelines (M100-ED29). Although the minimal inhibitory concentration of vancomycin was 4 μg/mL, the strain should be considered intrinsically resistant to vancomycin. Detection of *vanC* gene was not performed, and susceptibility to oxazolidinones was not assessed. Given that no high-level resistance to gentamicin was detected with a screening test on agar plates (the diameter of the inhibition circle was 22 mm), the antimicrobial regimen was changed to ampicillin (2 g every 4 h) and gentamicin on the fifth day of admission. Gentamicin was administered at 120 mg (equivalent to 3 mg/kg) for initial loading and then 45 mg (equivalent to 1 mg/kg) daily for maintenance. The dose of gentamicin was adjusted appropriately to maintain trough levels of < 1 μg/mL. The patient underwent aortic valve replacement (AVR) on the thirteenth hospital day. Vegetations were found to be attached to the ventricular side of all three aortic valve cusps (Fig. 3a). Histopathological examination of the resected valve showed nodular infiltration of lymphocytes and neutrophils into all the layers, with fibrin precipitation and necrosis and clusters of Gram-positive cocci on the surface, which confirmed the diagnosis of infective endocarditis. The day after AVR, the patient underwent emergency reoperation for cardiopulmonary arrest resulting from cardiac tamponade. The subsequent postoperative course was uneventful. Based on the negative results of both the blood culture taken on the fourth day of admission and excised aortic valve cultures, the patient was administered intravenous ampicillin and gentamicin for 6 weeks. Surveillance blood cultures on days 7, 11, and 27 remained negative. Renal failure and vestibulocochlear dysfunction were not observed. The patient was discharged 8 weeks after admission.

## Discussion and conclusions

Enterococci are estimated to cause approximately 10% of infective endocarditis. *E. faecalis* and *E. faecium* account for 95–98% of enterococcal endocarditis cases [2, 6]. The risk factors for enterococcal endocarditis include older age, male sex, having an intracardiac device, and infection in a healthcare-associated setting [6]. Treatment of enterococcal endocarditis is challenging because of its resistance to multiple drugs.

*E. casseliflavus* is known to cause blood stream infection, meningitis, and endophthalmitis in humans [5]. However, there are few reports that *E. casseliflavus* causes infective endocarditis, and there are no reports on the detailed clinical and treatment course, so the optimal treatment regimen and duration are unknown.

In our case, the patient met the pathological and clinical criteria for infective endocarditis based on the modified Duke criteria [2]. This indicates that *E. casseliflavus* also causes infective endocarditis.

According to the drug susceptibility pattern of enterococci in the United States, *E. casseliflavus* is susceptible to penicillin G and ampicillin [7]. It should be noted that *E. casseliflavus* is intrinsically resistant to vancomycin [8]. The *vanC-2* gene on the chromosome of *E. casseliflavus* alters the biosynthetic pathway of peptidoglycan, changing the _D-_Ala- _D-_Ala end, which is the binding site for vancomycin, to the _D-_Ala- _D-_Ser end [9]. This inhibits vancomycin binding. We should be aware that antimicrobial susceptibility tests of *E. casseliflavus* may report “susceptible” to vancomycin, as the vancomycin resistance resulting from *vanC* gene is of a low level. Although aminoglycosides are the cornerstone in the treatment of enterococcal infections, especially in infective endocarditis, approximately 18% of *E. casseliflavus* are reported to represent high-level resistance to gentamicin [10].

In general, single-agent treatment for enterococcal endocarditis is not recommended because approximately two-thirds of failures occur when penicillin is used as monotherapy [11]. The double-β-lactam regimen, or a combination of aminopenicillin and third-generation cephalosporin, showed a synergistic effect against *E. faecalis* [12]. This is considered to be caused by the partial saturation of penicillin-binding proteins (PBPs) 4 and 5 by aminopenicillin combined with the total saturation of PBPs 2 and 3 by third-generation cephalosporins. The profile of PBPs in *E. casseliflavus* is not well understood. Although the double-β-lactam regimen is one of the promising treatment options for endocarditis caused by *E. faecalis*, its effectiveness has not been proven in endocarditis caused by enterococci other than *E. faecalis* [13, 14]. Therefore, we referred to the treatment of endocarditis caused by a closely related species, *E. gallinarum*. In our search, we discovered five reported cases of infective endocarditis caused by *E. gallinarum* [15–19]. The treatment regimen was penicillin or ampicillin plus gentamicin in three cases [15–17], vancomycin in one case [18], and unknown in one case [19]. Although *E. casseliflavus* and *E. gallinarum* are genetically close and the resistance rate to ampicillin or aminoglycoside is similar, difference in resistance to erythromycin and vancomycin have been reported [20].

Based on the antimicrobial susceptibility pattern of isolated *E. casseliflavus* and these case reports, we chose ampicillin plus gentamicin. Although it is recommended that gentamicin should be administered in daily multiple divided doses in patients with enterococcal endocarditis [2], we administered gentamicin once daily because our patient had renal impairment. Older age and pre-existing renal disease are known to be risk factors for aminoglycoside nephrotoxicity [1]. Our case showed the possibility that some patients with these risk factors could complete the aminoglycoside-containing regimen without developing nephrotoxicity if the dose is appropriately adjusted using therapeutic drug monitoring. Alternative regimens for patients with multiple risk factors of nephrotoxicity include 2-weeks aminoglycoside or double-β-lactam regimen. However, further investigation is needed on such regimens for patients with endocarditis caused by *E. casseliflavus*.

According to several guidelines [2, 21], the duration of antimicrobial therapy in native valve enterococcal endocarditis depends on the duration of infection before diagnosis. Patients with < 3 months’ symptom duration can be successfully treated with 4 weeks of therapy, while patients with ≥3 months’ symptom duration can be successfully treated with 6 weeks of therapy. Although our patient developed acute respiratory failure, we opted for a 6-week regimen because the lumbago that had been observed for 5 months could have been due to vertebral osteomyelitis following infective endocarditis.

In conclusion, our case demonstrated that *E. casseliflavus* could cause infective endocarditis, which could be successfully treated with a 6-week regimen of ampicillin and gentamicin in combination with proper surgical treatment.

## Data Availability

Not applicable.

## Abbreviations

CCr: Creatinine clearance
MRI: Magnetic resonance imaging
MALDI-TOF: Matrix-assisted laser desorption/ionisation time-of-flight
MS: Mass spectrometry
AVR: Aortic valve replacement
PBP: Penicillin-binding protein
